# Supplementary material for: Complexome profiling of the Chlamydomonas psb28 mutant reveals TEF5 as an early PSII assembly factor
Source: Plant Cell. 2025 Mar 17;37(6):koaf055. doi: 10.1093/plcell/koaf055 (PMC12164590; doi:10.1093/plcell/koaf055)
Supplement: koaf055_Supplementary_Data [file koaf055_supplementary_data.zip › psb28_interactive.zip!/plots/intensity/qualityPlots/peptides/CGL51_Cre16.g663800.t1.2.html]

### Cre16.g663800.t1.2

Trivial name: CGL51  
SeqCoverage [median]: 4.50  
Mass: 43.35 kDa  
  
 

| Sequence | MissedCleavage | Mass [Da] | wtIntensity | muIntensity | Score |
| --- | --- | --- | --- | --- | --- |
| SLNDYK | 0 | 738.35 | 0.00 | 699.06 | 86.09 |
|  |  |  | 0.0 | 699.1 |  |
